# Effectiveness of diet and physical activity interventions amongst adults attending colorectal and breast cancer screening: a systematic review and meta-analysis

**DOI:** 10.1007/s10552-020-01362-5

**Published:** 2020-11-08

**Authors:** Samuel T. Orange, Kirsty M. Hicks, John M. Saxton

**Affiliations:** 1 School of Biomedical, Nutritional, and Sport Sciences, Faculty of Medical Sciences, The Medical School, Newcastle University, Newcastle upon Tyne, NE2 4HH UK; 2 Department of Sport, Exercise and Rehabilitation, Faculty of Health and Life Sciences, Northumbria University, Northumberland Building, Newcastle upon Tyne, NE1 8ST UK

**Keywords:** Cancer screening, Risk reduction, Health promotion, Physical activity, Diet

## Abstract

**Purpose:**

To estimate the effectiveness of tailored physical activity and dietary interventions amongst adults attending colorectal and breast cancer screening.

**Methods:**

Five literature databases were systematically searched to identify randomised controlled trials (RCTs) of tailored physical activity and/or dietary interventions with follow-up support initiated through colorectal and breast cancer screening programmes. Outcomes included markers of body fatness, physical activity, and dietary intake. Mean differences (MDs) or standardised mean differences (SMDs) with 95% confidence intervals (CIs) were pooled using random effects models.

**Results:**

Five RCTs met the inclusion criteria encompassing a total of 722 participants. Diet and physical activity interventions led to statistically significant reductions in body mass (MD − 1.6 kg, 95% CI − 2.7 to − 0.39 kg; *I*^2^ = 81%; low quality evidence), body mass index (MD − 0.78 kg/m^2^, 95% CI − 1.1 to − 0.50 kg/m^2^; *I*^2^ = 21%; moderate quality evidence), and waist circumference (MD − 2.9 cm, 95% CI − 3.8 to − 1.91; *I*^2^ = 0%; moderate quality evidence), accompanied by an increase in physical activity (SMD 0.31, 95% CI 0.13 to 0.50; *I*^2^ = 0%; low quality evidence) and fruit and vegetable intake (SMD 0.33, 95% CI 0.01 to 0.64; *I*^2^ = 51%; low quality evidence).

**Conclusion:**

There is low quality evidence that lifestyle interventions involving follow-up support lead to modest weight loss and increased physical activity and fruit and vegetable intake. Due to the modest intervention effects, low quality of evidence and small number of studies, further rigorously designed RCTs with long-term follow-up of modifiable risk factors and embedded cost–benefit analyses are warranted (PROSPERO ref: CRD42020179960).

**Electronic supplementary material:**

The online version of this article (10.1007/s10552-020-01362-5) contains supplementary material, which is available to authorized users.

## Introduction

Cancer is the second leading cause of death globally, accounting for an estimated 9.6 million deaths in 2018 [1]. In the United Kingdom (UK), one in two people will be diagnosed with cancer in their lifetime and cancer accounts for more than one quarter of all deaths [2]. However, it is estimated that 30–50% of all cancer cases are preventable [3]. The risk of cancer can be reduced through population screening by detecting localised cancers or premalignant lesions early to prevent metastatic progression [4]. The World Health Organisation Regional Office for Europe (WHO/Europe) advocate mass population screening for breast, colorectal and cervical cancers based on certain characteristics and contexts [5].

The risk of common cancers, such as colorectal and breast cancer, can also be reduced by modifying exposure to lifestyle risk factors, which include physical inactivity, being overweight or obese, and consuming an unhealthy diet [6]. Managing these risk factors also reduces the risk of developing other chronic conditions, including cardiovascular disease and type II diabetes mellitus [7]. The cancer screening setting has been identified as an ideal opportunity for health professionals to promote healthy lifestyle behaviours [8]. Approximately eight out of 10 adults attending colorectal, breast and cervical cancer screening clinics are willing to receive lifestyle advice [9], and physician endorsement is known to play a key role in the initiation of healthy behaviours [10]. Thus, cancer screening can provide a platform for the provision of lifestyle advice and for capitalising on the “teachable moment” [8] when some individuals are more amenable to engaging with risk-reducing interventions.

Strong epidemiological evidence suggests that colorectal and breast cancer incidences are related to lifestyle-modifiable risk factors, such as physical activity and body fatness [6, 11, 12], supporting the rationale for lifestyle interventions in the colorectal and breast cancer screening settings. For instance, the World Cancer Research Fund/American Institute for Cancer Research (WCRF/AICR) Continuous Update Project demonstrated that achieving the highest quartiles of total physical activity reduces the relative risk of colon and postmenopausal breast cancer by 20% and 13%, respectively [11]. Evidence presented in the same report shows that for every 5 kg/m^2^ increment in body mass index (BMI), the relative risks of colorectal and postmenopausal breast cancer are decreased by 5–12% [11]. In contrast, there is only limited evidence linking cervical cancer risk with body fatness [6, 11]. Data from randomised controlled trials (RCTs) also show that diet and physical activity interventions reduce markers of body fatness in populations that typically attend colorectal or breast cancer screening, such as overweight postmenopausal women [13]. Therefore, considering the current evidence-base, offering physical activity and diet advice within population-based colorectal and breast cancer screening programmes might yield meaningful reductions in the risk of developing these common cancers and other lifestyle-related diseases.

Patient information leaflets (PILs) have been widely used in healthcare settings to raise awareness of the relation between lifestyle and chronic disease, and typically provide general recommendations on physical activity, healthy eating and smoking cessation [14]. Whilst PILs have the potential to reach a wide audience in a cost-efficient manner, regular follow-up support with treatment providers might be required for health promotion interventions to be successful [15]. Importantly, tailoring lifestyle advice to each individual might also be a critical factor for changing the behaviour of screening patients [16], but follow-up support and personalised advice requires additional costs and personnel, which must be balanced with the potential health benefits.

To date, no studies have systematically evaluated evidence for the effectiveness of personalised lifestyle support in cancer screening settings as a means of informing best-practice guidance and identifying gaps in knowledge. Therefore, this systematic review and meta-analysis aimed to evaluate the effectiveness of tailored physical activity and dietary interventions involving follow-up support amongst adults attending colorectal and breast cancer screening. Outcomes included indices of body fatness, physical activity, dietary intake, and blood-borne biomarkers related to cancer or cardiometabolic disease risk.

## Methods

This systematic review was prospectively registered in the PROSPERO prospective register of systematic reviews (ref: CRD42020179960) and followed the Preferred Reporting Items for Systematic Reviews and Meta-Analyses (PRISMA) guidelines [17].

### Search strategy

An electronic search of PubMed, Web of Science, SportDiscus, CINAHL and Cochrane Central Register of Controlled Trials (CENTRAL) was conducted from inception to 5th April 2020. Table 1 presents the search string used in PubMed. Standard boolean operators (AND, OR) were used to concatenate the search terms. We also manually searched the reference lists and forward citations of included studies to identify potentially eligible studies.

**Table 1:** Search terms used in PubMed, CINAHL, and Cochrane CENTRAL

[MeSH Terms] ("Colorectal Neoplasms" OR "Breast Neoplasms" OR "Adenoma") AND "Early Detection of Cancer" AND ("Exercise" OR "Diet" OR “Nutrition Therapy” OR “Weight loss” OR "Risk Reduction Behavior" OR "Life Style" OR “Health Education”)
AND
[All Fields] (colorectal OR bowel OR colon OR rectal OR breast OR mammary) AND (cancer OR neoplas* OR malignant* OR carcinoma OR tumour OR adenoma* OR polyps) AND (“cancer screening” OR “breast screening” OR “bowel screening” OR “colorectal screening”) AND (“physical activity” OR exercise OR “interval training” OR “endurance training” OR “continuous training” OR “circuit training” OR “resistance training” OR “strength training” OR diet* OR “weight loss” OR “caloric restrict*” OR “calorie restrict*” OR “nutrition*” OR “lifestyle intervention” OR “lifestyle programme*” OR “lifestyle advice” OR “health promotion*”)
AND
[Filter] Journal Article AND English

### Inclusion criteria

Original research articles were included if they met the following inclusion criteria: (1) the study was an RCT published in a peer-reviewed Journal, (2) full-text was available in English, (3) participants were adults aged ≥ 18 years attending a population-based cancer screening programme for colorectal or breast cancer, (4) a tailored physical activity and/or dietary intervention was initiated through the cancer screening programme and involved ≥ 2 interactions with the intervention facilitator such as a healthcare professional or lifestyle counsellor, (5) the study included a control group that did not receive the intervention, (6) body mass or another lifestyle risk factor related to colorectal or breast cancer was assessed before and after the intervention, and (7) the follow-up period was at least four weeks. Studies were excluded if: (1) full-text was not available in English, (2) participants were not randomly allocated to an intervention or control group, (3) the intervention was not initiated through a colorectal or breast cancer screening programme, (4) the intervention involved < 2 interactions with the intervention facilitator or did not include a physical activity or dietary component, (5) a lifestyle risk factor was not assessed before or after the intervention, or (6) results were uninterpretable due to insufficient reporting of data.

WHO/Europe advocate mass population screening for breast, colorectal and cervical cancer based on certain characteristics and contexts [5]. We limited this review to breast and colorectal cancer screening programmes because the risk of developing colon and postmenopausal breast cancers is strongly related to lifestyle-modifiable risk factors, which include physical activity and body fatness [11, 12]. In addition, there is insufficient and suggestive evidence linking cervical cancer risk to physical activity and body fatness, respectively [6, 11]. For the purposes of this review, physical activity interventions could include the delivery of supervised exercise sessions, behaviour change counselling that aimed to increase levels of free-living habitual physical activity or structured exercise, or a combination of both. Similarly, dietary interventions could comprise structured diet plan, advice around weight loss, and/or guidance on healthy eating (e.g. increasing fruit and vegetable consumption). We defined an ‘interaction’ with the intervention facilitator as a face-to-face visit, telephone consultation, or an individually tailored letter/email. We operationalised the control group as a group of participants that received standard care only or standard care plus the recommendation to follow general physical activity and/or healthy eating guidelines, but did not receive the intended study intervention.

### Outcomes

Outcomes were lifestyle risk factors related to colorectal or postmenopausal breast cancer. The primary outcome was change in body mass. Secondary outcomes included other markers of body fatness in line with the WCRF/AICR Continuous Update Project [11] (BMI, waist circumference, waist to hip ratio, and body fat percentage), blood-borne biomarkers related to cancer (insulin, IGF axis, pro-inflammatory cytokines, adipokines, and sex hormones) or cardiometabolic disease (blood glucose, HbA1c, cholesterol, and triglycerides), dietary intake (fruit, vegetable, fibre, and alcohol consumption) and physical activity behaviour. Markers of body fatness and blood-borne biomarkers were required to be objectively evaluated by a study investigator, whereas dietary intake and physical activity behaviour could be objectively measured or self-reported by participants. All outcomes were continuous measures.

### Study selection

After the literature searches were completed, studies were collected into a single list in an Excel spreadsheet (Microsoft Corporation, Redmond, Washington, USA). The first author (STO) removed duplicates and screened the titles and abstracts to identify potentially eligible studies. Full-texts were obtained for all studies that appeared relevant or where there was any uncertainty. Subsequently, two authors (STO and KMH) independently examined each full-text manuscript to assess for eligibility. Any disagreements were resolved through discussion and/or consultation with the third author (JMS). Corresponding authors were contacted if a full-text manuscript could not be retrieved or to clarify aspects of the study in relation to the inclusion criteria.

### Data extraction

Data items extracted from each eligible study included: (1) participant characteristics, (2) sample size, (3) details of the intervention, (4) details of the control group, (5) length of follow-up, (6) details of the outcome measure(s), and (7) baseline, follow-up, and change score data for each outcome. In cases that studies had multiple follow-ups, we extracted data from the follow-up closest to the cessation of the intervention. If individual studies involved multiple relevant intervention groups, these were combined into a single group for the meta-analysis, as per Cochrane guidelines [18]. Study authors were contacted to obtain missing data wherever necessary. All data were extracted independently by two authors (STO and KMH) and tabulated in custom-designed Excel spreadsheets. Review authors cross-checked coding sheets and any conflicts between the reviewers were resolved in consensus meetings.

### Risk of bias

The revised Cochrane risk of bias tool for randomised trials (RoB 2) was used to judge the risk of bias for a specific outcome within each included study [19]. RoB 2 comprises five domains and a series of signalling questions about features of the RCT relating to: (1) the randomisation process, (2) deviations from intended interventions, (3) missing outcome data, (4) measurement of the outcome, and (5) selection of the reported result. Judgements for each domain and the overall risk of bias are expressed as ‘low’, ‘high’, or ‘some concerns’. As the primary outcome of this review, body mass was assessed for risk of bias. If this was not possible, self-reported physical activity was used as the outcome. Judgements were made independently by two authors (STO and KMH), with disagreements resolved firstly by discussion and then by consulting the third author (JMS). Small study effects (suggestive of publication bias) were explored with Egger’s test of the intercept [20] and by visually inspecting a funnel plot of all the effect estimates included in the review (regardless of the outcome measure) plotted against their corresponding sampling variance.

### Quality of evidence

We rated the quality of evidence for each meta-analysed outcome using the evidence grading system developed by the Grades of Recommendation, Assessment, Development, and Evaluation (GRADE) collaboration [21]. GRADE has four levels of evidence: very low, low, moderate and high. Our review only included RCTs (which start with a ‘high quality’ rating) and we downgraded the evidence for each outcome based on the following factors: (1) risk of bias, (2) inconsistency of results, (3) indirectness of evidence, (4) imprecision of results, and (5) publication bias [22]. The evidence was downgraded by one level if we judged that there was a *serious limitation* or by two levels if we judged there to be a *very serious limitation.* One review author (STO) initially graded the quality of evidence and then discussed the ratings with the other two authors (KMH, JMS). Any discrepancies were resolved through consensus. An overall GRADE quality rating was applied to the body of evidence by taking the lowest quality of evidence from all of the outcomes [23]. Judgements about evidence quality were justified and documented within a GRADE evidence profile (see Online Resource 1).

### Statistical analysis

Where two or more trials reported the same outcome using the same measurement scale, we performed a meta-analysis of mean differences (MDs) between intervention and control groups. Mean differences were calculated using the change score in each group (mean change from baseline to follow-up) and the SD of the change scores (SD_diff_). If the same measurement scale was not used, we pooled standardised mean differences (SMDs), which were calculated by dividing the MD by the pooled SD_diff_. Hedges' *g* correction was applied to the SMD to adjust for sample bias. Qualitative descriptors used to interpret the strength of the SMDs were based on Cohen’s (1988) criteria ( ±): trivial (< 0.2), small (0.2 to 0.49), moderate (0.5 to 0.79), and large (≥ 0.8).

If a study did not report SD_diff_ and it could not be retrieved from the corresponding author, it was estimated with the reported standard error (SE) or 95% confidence intervals (CIs) [18]. In cases that a study did not report any measures of variability (e.g., SD) or precision (e.g., SE or CI) alongside the within-group change scores, SD_diff_ was estimated using SDs at baseline (SD_baseline_) and post-intervention (SD_post_) in addition to the within-groups correlation coefficient (*r*) [18]:

$$\text{SDdiff }=\sqrt{{\text{SD}}_{\text{baseline}}^{2}+ {\text{SD}}_{\text{post}}^{2}-(2 \times r \times {\text{SD}}_{\text{baseline}} \times {\text{SD}}_{\text{post}})}$$

We followed guidelines by Rosenthal [24] to assume a conservative correlation of 0.7. Sensitivity analyses were performed with *r* = 0.5 and *r* = 0.9 to determine whether the results were robust to the use of imputed correlations. Meta-analyses were performed with a random effects model using the restricted maximum likelihood method to estimate between-study variance [25]. Studies were weighted according to the inverse of the sampling variance. When a meta-analysis included more than one outcome from the same study (such as if a study reported both objective and subjective measures of physical activity), effect estimates were nested within studies using a three-level meta-analytic structure to account for correlated effects [26].

Statistical heterogeneity between studies was evaluated with the Chi-squared test (χ^2^), and the proportion of variability in effect estimates due to heterogeneity rather than sampling error was estimated using the *I*^2^ statistic. Thresholds for the interpretation of *I*^2^ were in line with Cochrane recommendations: 0–40% (‘might not be important’), 30–60% (‘may represent moderate heterogeneity’), 50–90% (‘may represent substantial heterogeneity’), and 75–100% (‘considerable heterogeneity’) [27]. The importance of the observed *I*^2^ value was interpreted alongside its 95% CI and the *p*-value from the *χ*^2^ test [27]. We performed a Leave-One-Out analysis to assess whether removing an individual effect estimate from a meta-analysis influenced the pooled treatment effect or explained heterogeneity in cases of substantial or considerable heterogeneity. No meta-regressions were performed due to a low number of available studies [27]. We used SMDs for the funnel plot analysis so that all effect estimates were included in one plot. Statistical analyses were conducted using package meta in R version 3.6.3 (R Foundation for Statistical Computing, Vienna, Austria). Statistical significance was set at *p* < 0.05. Data are presented as pooled effect estimates with their corresponding 95% CIs. The search results, dataset, and statistical code are available on Open Science Framework [28].

## Results

### Study selection

**Fig. 1:**
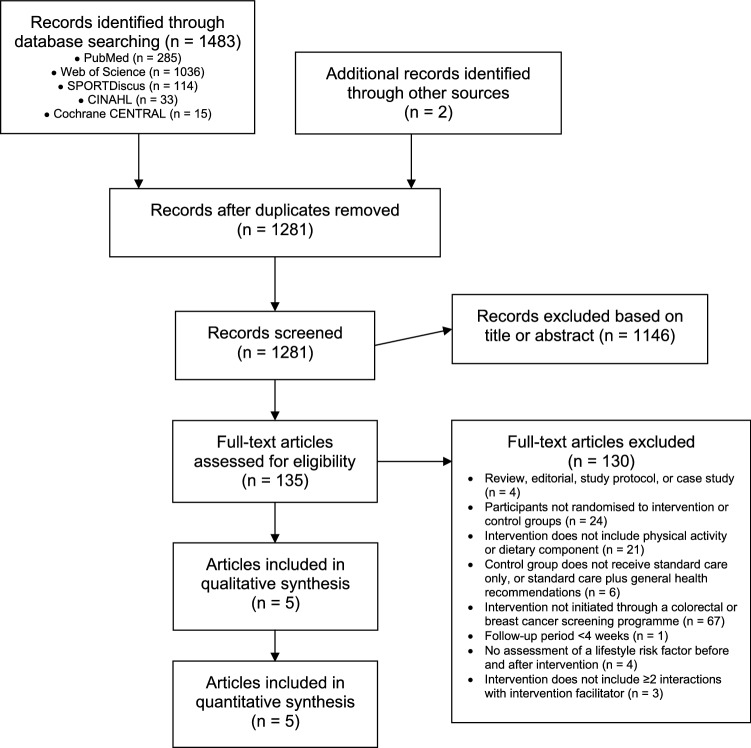
Preferred Reporting Items for Systematic Reviews and Meta-Analyses (PRISMA) flow diagram of the systematic search and included studies

The literature search yielded a total of 1,485 abstracts, of which 204 were duplicates (Fig. 1). After the screening of abstracts, 1,146 were removed and 135 full-texts were assessed for eligibility. A total of five studies met the inclusion criteria and were included in this review and meta-analysis.

### Included studies

An overview of study characteristics is presented in Table 2. The median sample size was 80 (range 25 to 329). Four of the five included studies were based in Scotland [29–32], with the remaining study based in Florence, Italy [33]. Three included studies involved adults having undergone a colonoscopy as part of a national colorectal cancer screening programme [29, 31, 32], whilst the other two included studies involved adults attending breast cancer screening by mammography [30, 33]. Three studies involved combined dietary and physical activity interventions [29–31], one study involved a physical activity-only intervention [32], and one study involved three intervention groups consisting of diet-only, physical activity-only, and combined interventions [33]. The median number of interactions with an intervention facilitator was 12 (range 4 to 125). Two studies had a final follow-up at 3-months [30, 31], two studies had a 12-month follow-up [29, 32], and one study had a 24-month follow-up [33].

**Table 2:** Description of included studies

								Main outcomes included in the review			
Study	Cancer screening	*N*^a^	Follow-up (months)	Overview	No. of interactions	Intervention adherence	Control group	Body fatness	Dietary intake	Physical activity	
										Objective	Self-report
Anderson et al. [29]	Colorectal	I: 163 C: 166	3 and 12	Diet and PA advice delivered by lifestyle counsellor over 12 months	*n* = 12 3 × 1 h face-to-face visits plus 9 × 15 min monthly telephone consultations	97% attended all 3 face-to-face visits, 59% completed all 9 telephone calls	BHF weight loss leaflet	Body mass BMI WC	Fruit and vegetable Fibre Alcohol	Waist-worn ACC Total MVPA (min day^−1^)	-
Anderson et al. [30]	Breast	I: 40 C: 40	3	Diet and PA advice delivered by lifestyle counsellor over 3 months	*n* = 7 1 × 1 h face-to-face visit plus 6 × 15 min fortnightly telephone consultations	93% attended face-to-face visit, 78% completed all 6 telephone calls	WCRF breast cancer prevention leaflet	Body mass BMI WC	Fruit Vegetable Fibre Alcohol	–	IPAQ-SF Total walking plus MVPA (MET min wk^−1^)
Caswell et al. [31]	Colorectal	I: 32 C: 30	3	Diet and PA advice delivered by researcher over 3 months	*n* = 4 1 × 2 h face-to-face visit plus 3 × mailings	NR	Assessments only	–	Fruit and vegetable Fibre	–	SPAQ-2 Total MVPA (min day^−1^)
Lewis et al. [32]	Colorectal	I: 12 C: 13	6 and 12	PA advice and supervised exercise delivered by exercise specialist over 6 months	*n* = 48 36 supervised exercise sessions (30 min aerobic exercise @ 65–85% MHR plus 10–15 min RT, 1–2 × /week) plus 12 weekly behaviour change workshops	Mean attendance: 72% for exercise sessions and 65% for behaviour change workshops	Assessments only	Body mass BMI WC	–	Arm-worn ACC Total MVPA (min wk^−1^)	IPAQ-LF Total MVPA (min wk^−1^)
Masala et al. [33]	Breast	I_1_: 57 I_2_: 54 I_3_: 55 C: 60	24	I_1_: Diet advice delivered over 24 months I_2_: PA advice and supervised exercise delivered by PA expert over 24 months I_3_: Diet and PA advice and supervised exercise delivered over 24 months	I_1_: *n* = 15 1 × face-to-face visit plus 6 × group meetings and 8 × cooking classes I_2_: *n* = 110 97 supervised exercise sessions (60 min, 1 × /week) plus 1 × face-to-face visit, 6 × group meetings, and 6 × group walks I_3_: *n* = 125 Combined I_1_ and I_2_	I_1_: NR I_2_: Mean attendance to exercise sessions was 57% I_3_: Mean attendance to exercise sessions was 46%	General healthy diet and PA advice according to WCRF 2007 guidelines	Body mass	Fruit and vegetable	-	EPIC-PAQ Total leisure-time PA (MET hr wk^−1^)

*ACC* accelerometer, *BHF* British Heart Foundation, *BMI* body mass index, *C* control group, *EPIC-PAQ* European Prospective Investigation into Cancer and Nutrition—Physical Activity Questionnaire, *I* intervention group, *IPAQ* International Physical Activity Questionnaire, *MHR* maximum heart rate, *LF* long form, *MVPA* moderate- to vigorous-intensity physical activity, *NR* not reported, *PA* physical activity, *SF* short form, *SPAQ-2* Scottish Physical Activity Screening Questionnaire-2, *WC* waist circumference, *WCRF* World Cancer Research Fund

^a^Number of participants included in the analysis of the primary outcome

### Risk of bias

**Fig. 2:**
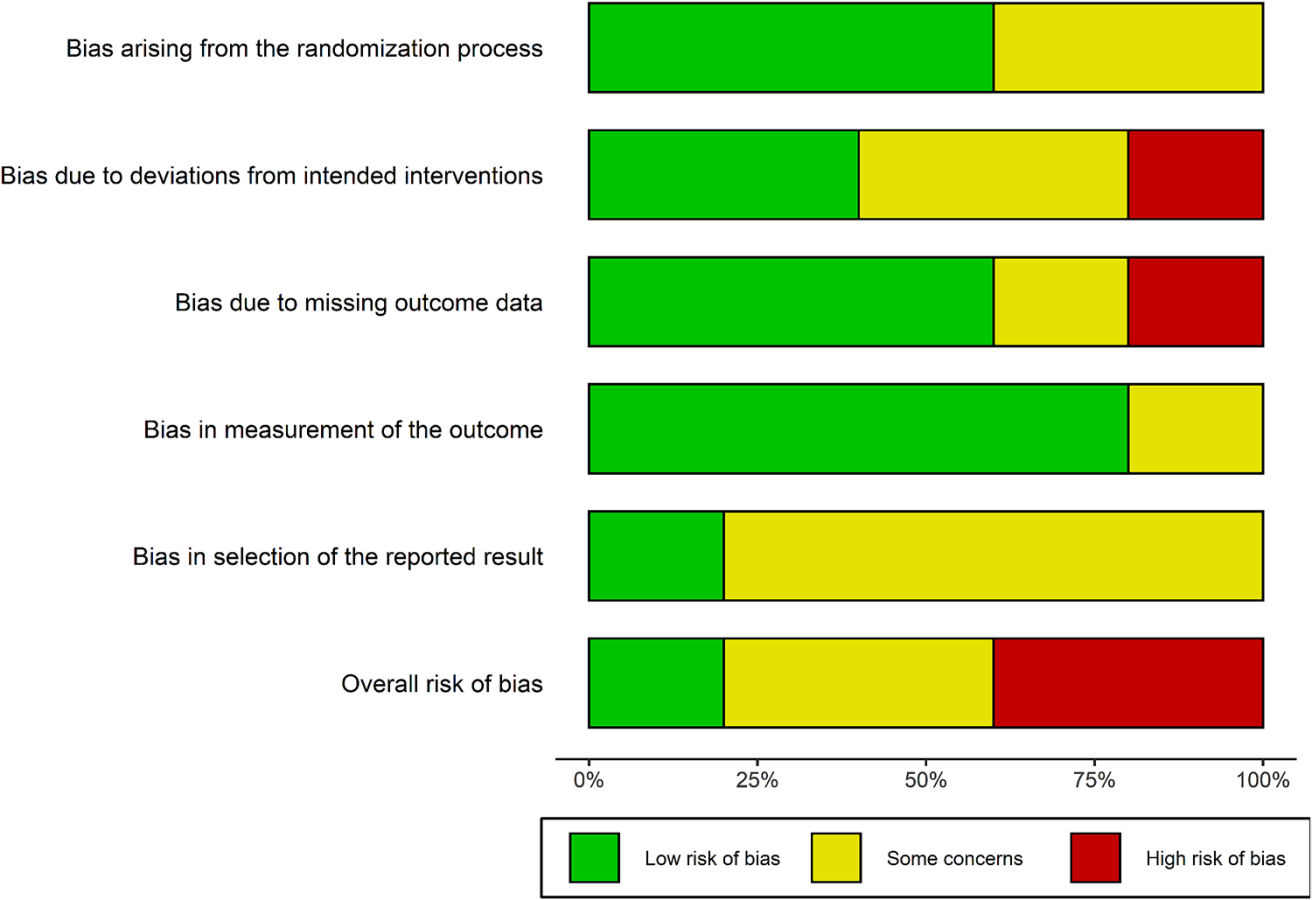
Summary of review authors’ risk of bias judgement for each domain across all included studies using the revised Cochrane risk of bias tool for randomised trials

Of the five RCTs included in the review, one study was judged to have an overall low risk of bias [29], two studies were considered to have a high overall risk of bias [30, 32] and two were judged to raise some concerns overall [31, 33] (Fig. 2). Judgements for each domain in each included study are presented in Online Resource 2. Visual inspection of the funnel plot showed that the treatment effects were symmetrically distributed around the overall pooled effect size (see Online Resource 3). In addition, Egger’s test of the intercept showed that sampling variance did not statistically mediate the overall effect estimate (β =  − 0.15; 95% CI − 3.2 to 2.9, *p* = 0.92).

### Outcomes

#### Body mass

**Fig. 3:**
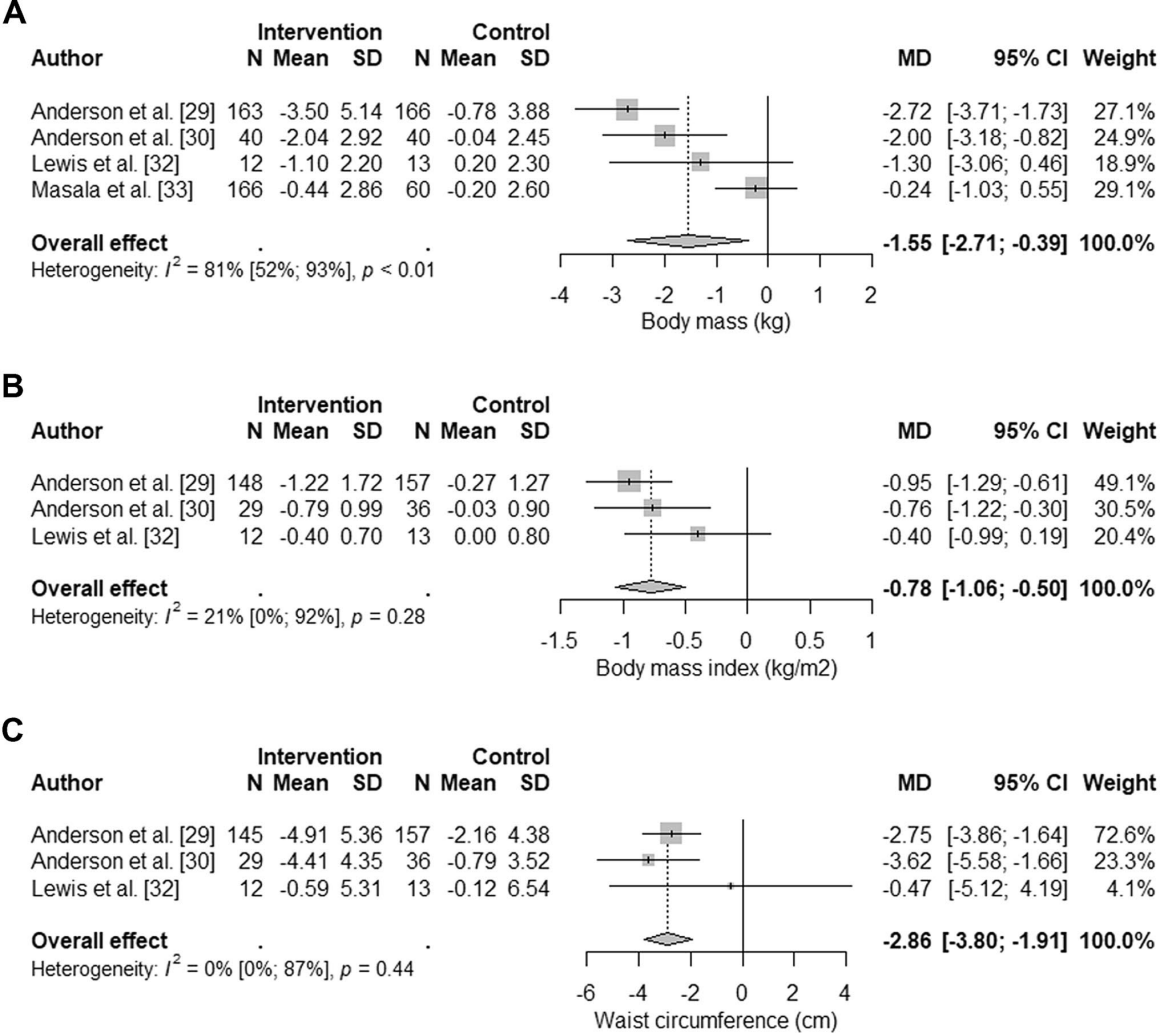
Forest plot of the results from random effects meta-analyses on body mass (panel A), body mass index (panel B), and waist circumference (panel C). Data are presented as mean difference (MD) between intervention and control groups with corresponding 95% confidence interval (CI)

**Fig. 4:**
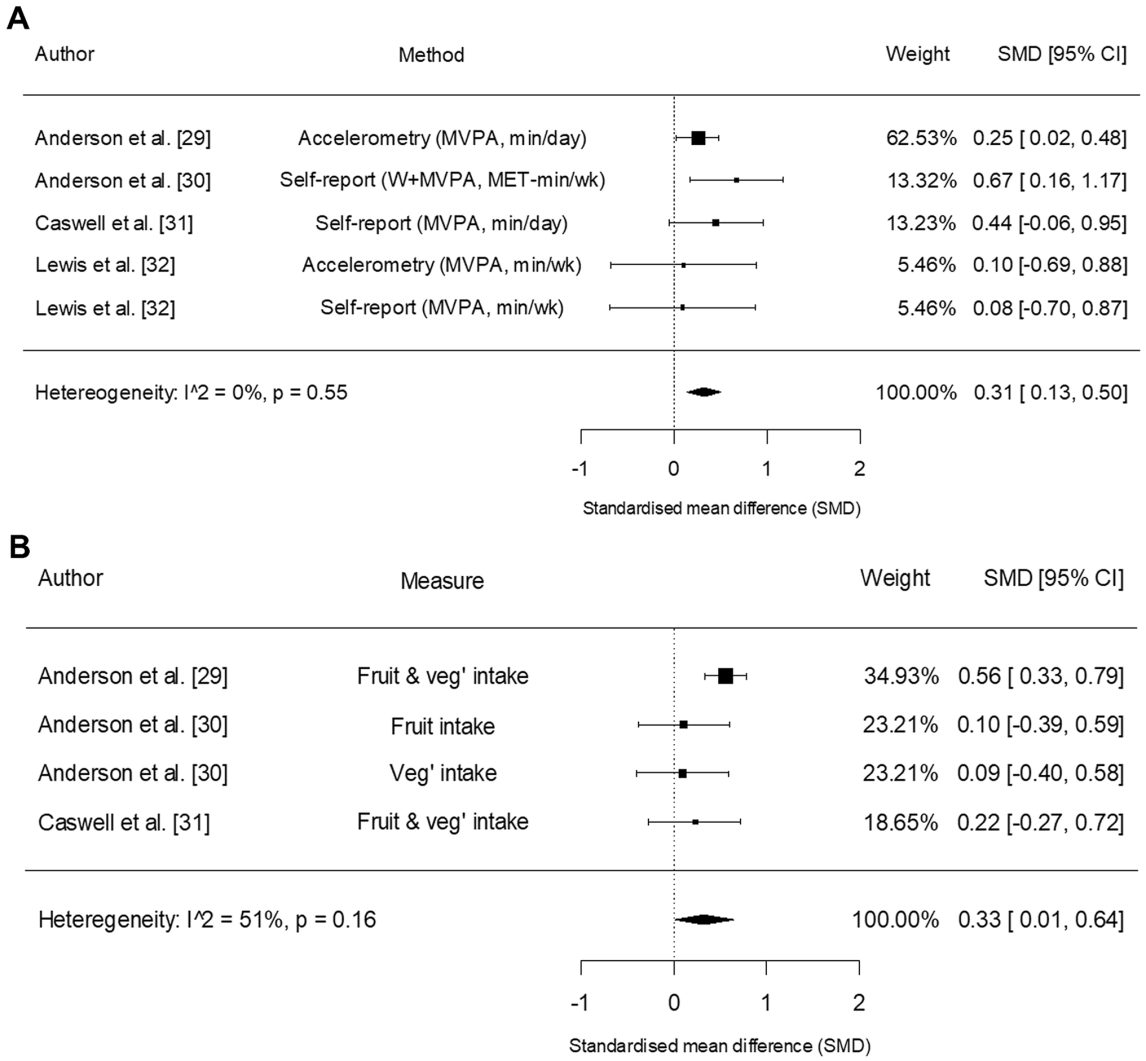
Forest plot of the results from random effects meta-analyses on physical activity (panel A) and fruit and vegetable intake (panel B). Data are presented as mean difference (MD) between intervention and control groups with corresponding 95% confidence interval (CI). *MVPA* moderate- to vigorous-intensity physical activity, *W* walking

The pooled results of four RCTs [29, 30, 32, 33] consisting of 660 participants showed a statistically greater weight loss following the intervention compared with controls (MD − 1.6 kg, 95% CI − 2.7 to − 0.39 kg; *p* = 0.009; low quality evidence) (Fig. 3, 4). There was evidence of considerable between-study heterogeneity (*I*^2^ = 81%). Removal of one RCT from the meta-analysis [33] explained almost all of the heterogeneity (*I*^2^ = 7%). Omitting individual studies also influenced the meta-analysis results so that the 95% CI crossed the line of no effect (see Online Resource 4).

#### BMI

The combined results of three RCTs [29, 30, 32] involving 395 participants showed a greater reduction in BMI in the intervention groups compared with controls (MD − 0.78 kg/m^2^, 95% CI − 1.1 to − 0.50 kg/m^2^; *p* < 0.001; moderate quality evidence). The magnitude of the between-study heterogeneity was not important (*I*^2^ = 21%) and the meta-analytic result was robust to omitting individual studies (see Online Resource 4).

#### Waist circumference

Based on pooled data from three RCTs [29, 30, 32] with 392 participants, diet and physical activity interventions statistically reduced waist circumference compared with control groups (MD − 2.9 cm, 95% CI − 3.8 to − 1.91; *p* < 0.001; moderate quality evidence). Between-study heterogeneity was not important (*I*^2^ = 0%) and the pooled MD remained statistically significant after omitting individual studies (see Online Resource 4).

#### Physical activity

All five included RCTs evaluated physical activity. One study objectively measured physical activity via accelerometery [29], three studies employed self-report questionnaires [30, 31, 33], and one study used both objective (accelerometery) and self-report measures [32]. Data from one RCT were insufficient to pool [33]. A meta-analysis of the remaining four RCTs [29–32] consisting of 440 participants showed a statistically significant increase in physical activity in the intervention groups compared with controls (SMD 0.31, 95% CI 0.13 to 0.50; *p* = 0.001; low quality evidence). The magnitude of heterogeneity was not important (*I*^2^ = 0%) and the overall treatment effect was robust to removal of individual studies (see Online Resource 4).

#### Fruit and vegetable intake

Four RCTs assessed self-reported fruit and vegetable intake, either using the Dietary Instrument for Nutrition Education (DINE) [29–31] or the Food Frequency Questionnaire [33]. Data reported in one study [33] were insufficient to include in the meta-analysis. Pooled data from the three remaining RCTs [29–31] involving 432 participants showed a statistically significant increase in favour of the intervention compared with control (SMD 0.33, 95% CI 0.01 to 0.64; *p* = 0.041; low quality evidence). The magnitude of between-study heterogeneity was moderate (*I*^2^ = 51%). Removing individual studies influenced the results so that the 95% CI crossed zero (see Online Resource 4).

#### Fibre intake

Three RCTs [29–31] used DINE to evaluate fibre intake. The DINE fibre score ranges from 3–88 (arbitrary units) with a score of less than 30 (low) corresponding to a fibre intake of ≤ 20 g/day, and a score of more than 40 (high) corresponding to ≥ 30 g/day. Pooling the results of these three RTCs with a total of 432 participants showed no statistical difference between intervention and control groups (MD 4.3 arbitrary units, 95% CI − 3.0, to 11.5 arbitrary units; *p* = 0.25; low quality evidence) (see Online Resource 5). There was evidence of considerable between-study heterogeneity (*I*^2^ = 92%), although this was completely explained by removing one RCT [31] from the meta-analysis (*I*^2^ = 0%; see Online Resource 4).

#### Alcohol consumption

Two RCTs evaluated alcohol intake using either a 7-day recall [30] or questions from the Alcohol Use Disorders Inventory Test [29]. Insufficient data presented in one of the RCTs [29] precluded a meta-analysis.

#### Other outcomes

Outcomes related to waist to hip ratio [32], body fat percentage [32], and blood-borne biomarkers [29] were only reported by individual studies and therefore the data were insufficient to pool.

### Sensitivity analyses

The within-groups SD_diff_ was unavailable from extraction in two RCTs [29, 31] for outcomes on physical activity, fibre intake, and fruit and vegetable intake. Estimating SD_diff_ assuming *r* = 0.5 instead of *r* = 0.7 did not substantially influence the conclusions of the meta-analyses. However, assuming *r* = 0.9 changed the results for the meta-analysis on fruit and vegetable intake in such a way that the 95% CI crossed the line of no effect (see Online Resource 6).

## Discussion

This is the first study to systematically review the impact of initiating diet and physical activity interventions within colorectal and breast cancer screening programmes. The main findings were that lifestyle interventions involving follow-up support led to modest weight loss and increased physical activity and fruit and vegetable intake compared with usual care. However, the clinical meaningfulness of these findings is uncertain due to the small intervention effects, low number of eligible RCTs, and low overall quality of evidence.

WHO/Europe advocate mass population screening for breast and colorectal cancer to reduce the cancer burden [5]. Cancer screening has been described as a “teachable moment” and an opportune time to promote risk-reducing behaviours [8]. Indeed, eight out of 10 adults attending colorectal, breast and cervical cancer screening clinics are willing to receive lifestyle advice [9]. Modifying or avoiding exposure to lifestyle risk factors (including obesity, physical inactivity, dietary factors, and alcohol consumption) decreases the risk of developing colorectal and postmenopausal breast cancer [6], as well as other non-communicable diseases such as cardiovascular disease and type II diabetes mellitus [7]. Thus, combining cancer screening with lifestyle interventions may be a key strategy for system-wide disease prevention.

Our meta-analysis of four RCTs showed that diet and/or physical activity interventions led to modest weight loss amongst adults attending colorectal or breast cancer screening. We also found statistically significant reductions in other anthropometric markers of body fatness, including BMI and waist circumference. These are key findings because weight loss is recommended for adults with a BMI above 24.9 kg/m^2^ to reduce the risk of developing common cancers, including colorectal and postmenopausal breast cancer [11]. Whilst the minimum clinically important weight loss for impacting cancer risk is unknown, the American College of Cardiology/American Heart Association (ACC/AHA) suggest an average weight loss of ≥ 2.5 kg is clinically significant for reducing type II diabetes risk [34]. Others consider weight change of ≥ 5% to be clinically significant for cardiovascular disease risk [35, 36]. The pooled weight loss from our meta-analysis (1.6 kg) represents a ≈2.1% decrease from baseline values, which is below these thresholds. The upper 95% CI of the pooled effect (2.7 kg) also does not represent a ≥ 5% weight loss, suggesting the highest weight loss compatible with the data included in this review still may not be meaningful. Similarly, the pooled MD in waist circumference (− 2.9 cm) may not be clinically important [37]. Therefore, current evidence suggests that embedding diet and physical activity advice within the cancer screening setting results in weight loss; however, the magnitude of weight loss might be below the threshold required to elicit meaningful health benefits.

In addition to the modest intervention effects, the quality of evidence for body mass was low. This was primarily due to risk of bias within individual studies, and because the treatment effect for body mass showed considerable heterogeneity (*I*^2^ = 81%) and was sensitive to the omission of individual studies. Indeed, removing either Anderson et al. [29] or Anderson et al. [30] from the meta-analysis resulted in the MD (95% CI) crossing the line of no effect, raising questions about the robustness of the overall pooled effect. In addition, removing one RCT [33] almost entirely explained the between-study heterogeneity (*I*^2^ = 7%). Further high-quality evidence is therefore required to increase our confidence in the estimated treatment effect. Accordingly, the ongoing ActWELL trial [38] is assessing the impact of lifestyle interventions on weight loss in women attending breast cancer screening and will make an important contribution to this body of evidence.

The diet and physical activity interventions led to small increases in moderate- to vigorous-intensity physical activity compared to controls. Physical activity is inversely associated with the risk of colon and postmenopausal breast cancer, independent of body fatness [12, 39]. Intervention studies also show that regular aerobic exercise can improve glycaemic control, insulin action and blood lipid profile in the absence of weight loss [40]. Thus, strategies to increase physical activity could be an important component of lifestyle interventions in colorectal or breast cancer screening settings, independent of weight loss. However, the intervention effect was small (SMD = 0.31) and the quality of evidence for physical activity was low, partly because it was assessed using a combination of objective and self-reported methods. There is often discordance between objective and self-report measures of physical activity [41], with self-report methods being limited by poor validity for measuring lifestyle physical activities, participant response bias and misunderstanding of questions [42]. The AHA recommend that when a high level of accuracy is required and resources are available, researchers should assess physical activity with objective measures such as accelerometery [42].

We also observed a small increase in self-reported fruit and vegetable intake following the diet and physical activity interventions. However, similar to the body mass outcome, omitting individual studies from the meta-analysis changed the results so that the 95% CI of the treatment effect crossed zero. In addition, there was no evidence for an effect on fibre intake and there were unsufficient data to pool effect estimates on alcohol consumption.

All RCTs in this review included a tailored diet and physical activity intervention arm that involved follow-up support (≥ 2 interactions with the intervention facilitator). This is in contrast to PIL interventions, which comprise general physical activity and dietary advice without reinforcement or follow-up support [14]. Whilst standard PILs are less expensive than tailored interventions and are widely used as standard care throughout the healthcare sector, RCTs have shown that they are not effective for eliciting behaviour change in adults attending colorectal cancer screening [43, 44] or those at high-risk for cardiovascular disease [45]. Previous research with adults who are overweight or obese also show that extended care in the form of continued contact with the treatment provider (typically once or twice per month) improves the maintenance of lost weight [15, 46, 47]. Nevertheless, for implementation into standard care, the benefits of personalised lifestyle interventions with follow-up support must outweigh the cost of such provision within resource-constrained healthcare systems. As previously discussed, the modest intervention effects found in this review may not, on average, elicit meaningful health benefits in cancer screening patients, which suggests that personalisation of lifestyle advice and continued support may not be economically worthwhile for service providers. Further trials with embedded cost–benefit analyses are clearly warranted.

This review has some limitations. At the study level, only one RCT [29] included in the review was judged to have a low risk of bias. Common issues included a lack of information about allocation concealment [31, 33], participant retention of < 85% [30–32], the absence of ‘intention to treat’ analyses [31–33], and a lack of prospective registration on a public trials registry [30–33]. In addition, two RCTs only followed-up outcomes for 3-months, which limits our understanding of the long-term effectiveness of lifestyle interventions.

A limitation at the review-level is that we restricted the literature search to English-language RCTs published in peer-reviewed Journals, and therefore might have missed some relevant studies in the grey literature. In addition, the small number of RCTs included in the review prevented us from performing meta-regressions or subgroup analyses to further explore sources of heterogeneity in the treatment effects, although we were largely able to explain heterogeneity with the Leave-One-Out sensitivity analysis. The small number of studies also precluded us from creating a funnel plot for each outcome; instead, we combined all outcomes together in one funnel plot, which is suboptimal because different outcomes may have different risks of bias. Furthermore, the results of this review were based on pooled data from RCTs in Scotland and Italy, which may not be generalisable to cancer screening programmes in other countries. Finally, there were minor deviations from the pre-registered protocol [28], including extracting outcome data on alcohol consumption and blood-borne biomarkers, which was not initially stipulated in the protocol. Following peer-review feedback, we also used the Cochrane RoB 2 to evaluate risk of bias rather than the pre-specified Physiotherapy Evidence Database scale.

In conclusion, there is low quality evidence that tailored diet and physical activity interventions involving follow-up support lead to modest weight loss, increased physical activity, and increased fruit and vegetable intake amongst adults attending colorectal and breast cancer screening. Due to the modest intervention effects, low quality of evidence and small number of eligible studies, further rigorously designed RCTs with long-term follow-up of modifiable risk factor outcomes and embedded cost–benefit analyses are warranted.

## Electronic supplementary material

Below is the link to the electronic supplementary material.

Electronic supplementary material 1 (PDF 103 kb)

Electronic supplementary material 2 (PDF 76 kb)

Electronic supplementary material 3 (PDF 18 kb)

Electronic supplementary material 4 (DOCX 31 kb)

Electronic supplementary material 5 (PDF 20 kb)

Electronic supplementary material 6 (PDF 32 kb)

## Data Availability

The search results and datasets from this review are available in the Open Science Framework repository [https://bit.ly/2Zeq4xD]. The statistical code from this review is available in the Open Science Framework repository [https://bit.ly/2Zeq4xD].
