# Supplementary material for: Effectiveness of diet and physical activity interventions amongst adults attending colorectal and breast cancer screening: a systematic review and meta-analysis
Source: Cancer Causes Control. 2020 Nov 8;32(1):13–26. doi: 10.1007/s10552-020-01362-5 (PMC7796884; doi:10.1007/s10552-020-01362-5)
Supplement: Supplementary file 1 — Electronic supplementary material 1 (PDF 103 kb) [file 10552_2020_1362_MOESM1_ESM.pdf]

**Electronic supplementary material 1.** GRADE evidence profile

| Summary of findings | | | | Quality assessment | | | | | |
| --- | --- | --- | --- | --- | --- | --- | --- | --- | --- |
| Outcome | No. of participants (studies) | Pooled treatment effect (95% CI) | $I^2$ | Risk of bias | Inconsistency | Indirectness | Imprecision | Publication bias† | Quality rating |
| Body mass (kg) | 660 (4) | -1.6 (-2.7, -0.39) | 81% | Serious limitations[a] | Serious inconsistency[b] | No serious indirectness | No serious imprecision[c] | Undetected | Low |
| Body mass index (kg/m$^2$) | 395 (3) | -0.78 (-1.1, -0.50) | 21% | Serious limitations[d] | No serious inconsistency | No serious indirectness | No serious imprecision[c] | Undetected | Moderate |
| Waist circumference (cm) | 392 (3) | -2.9 (-3.8, -1.9) | 0% | Serious limitations[d] | No serious inconsistency | No serious indirectness | No serious imprecision[c] | Undetected | Moderate |
| Physical activity | 440 (4) | 0.31 (0.13, 0.50) | 0% | Serious limitations[a] | No serious inconsistency | Serious indirectness[e] | No serious imprecision[c] | Undetected | Low |
| Fruit and vegetable intake | 432 (4) | 0.33 (0.01, 0.63) | 51% | Serious limitations[a] | Serious inconsistency[f] | No serious indirectness | No serious imprecision[c] | Undetected | Low |
| Fibre intake | 432 (3) | 4.3 (-3.0, 11.5) | 92% | Serious limitations[a] | No serious inconsistency[g] | No serious indirectness | Serious imprecision[h] | Undetected | Low |

[a]Most of the evidence comes from studies with a crucial limitation in one domain of Cochrane risk of bias tool for randomized trials (RoB 2) or some limitations in multiple domains.

[b]The proportion of between-study variance due to heterogeneity was considerable and removal of individual studies (via a Leave-One-Out analysis) influenced the meta-analysis results so that the 95% CI crossed the line of no effect.

[c]The total number of participants exceeds to Optimal Information Size (OIS)* criterion and the 95% confidence interval (CI) excludes zero, and therefore the evidence was not downgraded.

[d]Most of the evidence comes from studies with a crucial limitation in one domain of RoB 2.

[e]Measures of physical activity included both objective and self-reported measures. Self-reported measures may be suboptimal and are likely to be prone to response bias compared with objectively-measured physical activity.

[f]The proportion of between-study variance due to heterogeneity was moderate and removal of individual studies (via a Leave-One-Out analysis) influenced the meta-analysis results so that the 95% CI crossed the line of no effect.

[g]The proportion of between-study variance due to heterogeneity was considerable, however, removal of one study reduced $I^2$ to 0%. Therefore, we did not downgrade the evidence because the considerable heterogeneity was entirely explained by one study.

[h]The total number of participants exceeds to OIS criterion, but the 95% CI crossed zero and the upper CI bound failed to exclude the minimum clinically important difference (MCID; considered to be 5 arbitrary units).

*For mean differences, the OIS was calculated using $\alpha = 0.05$, $\beta = 0.20$, an independent *t*-test, and by identifying an MCID and extracting the SDs associated with the relevant outcome from studies included in the meta-analysis (see Table below). For standardised mean differences, the minimum important difference for calculation of the OIS was 0.5 SDs.
†The low number of studies precluded the assessment of publication bias for each outcome using funnel plot or regression-based methods. Therefore, we combined all effect estimates across all outcomes in one funnel plot by calculating standardised mean differences (and corresponding sampling variances) and nesting effect sizes within each study. As a result, the assessment of publication bias relates to all outcomes included in the review, not each individual outcome.

**Minimum clinically important difference (MCID) and standard deviations (SDs) used to inform calculation of the Optimal Information Size (OIS) for outcomes reported as a mean difference**

| Outcome | MCID | Most conservative SD from relevant studies | OIS |
|---|---:|---:|---:|
| Body mass (kg) | 2.5 kg | 4.5 kg | 106 |
| Body mass index ($kg/m^2$) | 0.5 $kg/m^2$ | 1.5 $kg/m^2$ | 292 |
| Waist circumference (cm) | 3 cm | 6.0 cm | 128 |
| Fibre intake | 5 arbitrary units | 8.7 arbitrary units | 100 |
