# Supplementary material for: Effectiveness of diet and physical activity interventions amongst adults attending colorectal and breast cancer screening: a systematic review and meta-analysis
Source: Cancer Causes Control. 2020 Nov 8;32(1):13–26. doi: 10.1007/s10552-020-01362-5 (PMC7796884; doi:10.1007/s10552-020-01362-5)
Supplement: Supplementary file 4 — Electronic supplementary material 4 (DOCX 31 kb) [file 10552_2020_1362_MOESM4_ESM.docx]

| **Electronic supplementary material 4.** Leave-one-out sensitivity analyses on each meta-analysed outcome | | | |
| --- | --- | --- | --- |
|  | Pooled treatment effect (95% CI) | *p*-value | *I^2^* |
| **Body mass (MD, kg)** |  |  |  |
| **All studies included** | **-1.6 (-2.7, -0.39)** | **0.009** | **81%** |
| Omitting Anderson et al. [29] | -1.1 (-2.2, 0.07) | 0.066 | 68% |
| Omitting Anderson et al. [30] | -1.4 (-3.0, 0.14) | 0.074 | 87% |
| Omitting Lewis et al. [32] | -1.6 (-3.1, -0.13) | 0.033 | 88% |
| Omitting Masala et al. [33] | -2.2 (-3.0, -1.50) | <0.001 | 7% |
| **BMI (MD, kg/m^2^)** |  |  |  |
| **All studies included** | **-0.78 (-1.06, -0.50)** | **<0.001** | **22%** |
| Omitting Anderson et al. [29] | -0.62 (-0.99, -0.26) | 0.001 | 0% |
| Omitting Anderson et al. [30] | -0.73 (-1.3, -.20) | 0.007 | 60% |
| Omitting Lewis et al. [32] | -0.88 (-1.2, -0.61) | <0.001 | 0% |
| **Waist circumference (MD, cm)** |  |  |  |
| All studies included | -2.9 (-3.8, -1.9) | <0.001 | 0% |
| Omitting Anderson et al. [29] | -2.8 (-5.5, -0.04) | 0.047 | 33% |
| Omitting Anderson et al. [30] | -2.6 (-3.7, -1.5) | <0.001 | 0% |
| Omitting Lewis et al. [32] | -3.0 (-3.9, -2.0) | <0.001 | 0% |
| **Fibre intake (MD, arbitrary units)** |  |  |  |
| **All studies included** | **4.3 (-3.0, 11.5)** | **0.25** | **92%** |
| Omitting Anderson et al. [29] | 6.6 (-3.7, 17.0) | 0.21 | 93% |
| Omitting Anderson et al. [30] | 5.8 (-5.9, 17.6) | 0.33 | 96% |
| Omitting Caswell et al. [31] | 0.32 (-1.2, 1.8) | 0.68 | 0% |
| **Physical activity (SMD)** |  |  |  |
| **All studies included** | **0.31 (0.13, 0.50)** | **0.001** | **0%** |
| Omitting Anderson et al. [29] | 0.42 (0.10, 0.73) | 0.009 | 6% |
| Omitting Lewis et al. [32] | 0.33 (0.13, 0.53) | 0.001 | 5% |
| Omitting Lewis et al. [32] | 0.33 (0.13, 0.54) | 0.001 | 5% |
| Omitting Anderson et al. [30] | 0.26 (0.06, 0.46) | 0.010 | 0% |
| Omitting Caswell et al. [31] | 0.30 (0.08, 0.53) | 0.009 | 9% |
| **Fruit/vegetable intake (SMD)** |  |  |  |
| **All studies included** | **0.33 (0.01, 0.64)** | **0.041** | **51%** |
| Omitting Anderson et al. [29] | 0.14 (-0.15, 0.42) | 0.34 | 0% |
| Omitting Anderson et al. [30] | 0.36 (0.05, 0.67) | 0.025 | 47% |
| Omitting Anderson et al. [30] | 0.36 (0.06, 0.67) | 0.020 | 45% |
| Omitting Caswell et al. [31] | 0.35 (-0.10, 0.80) | 0.13 | 67% |
| 95% CI = 95% confidence interval; MD = mean difference; SMD = standardised mean difference | | | |
