# Supplementary material for: Effectiveness of diet and physical activity interventions amongst adults attending colorectal and breast cancer screening: a systematic review and meta-analysis
Source: Cancer Causes Control. 2020 Nov 8;32(1):13–26. doi: 10.1007/s10552-020-01362-5 (PMC7796884; doi:10.1007/s10552-020-01362-5)
Supplement: Supplementary file 5 — Electronic supplementary material 5 (PDF 20 kb) [file 10552_2020_1362_MOESM5_ESM.pdf]

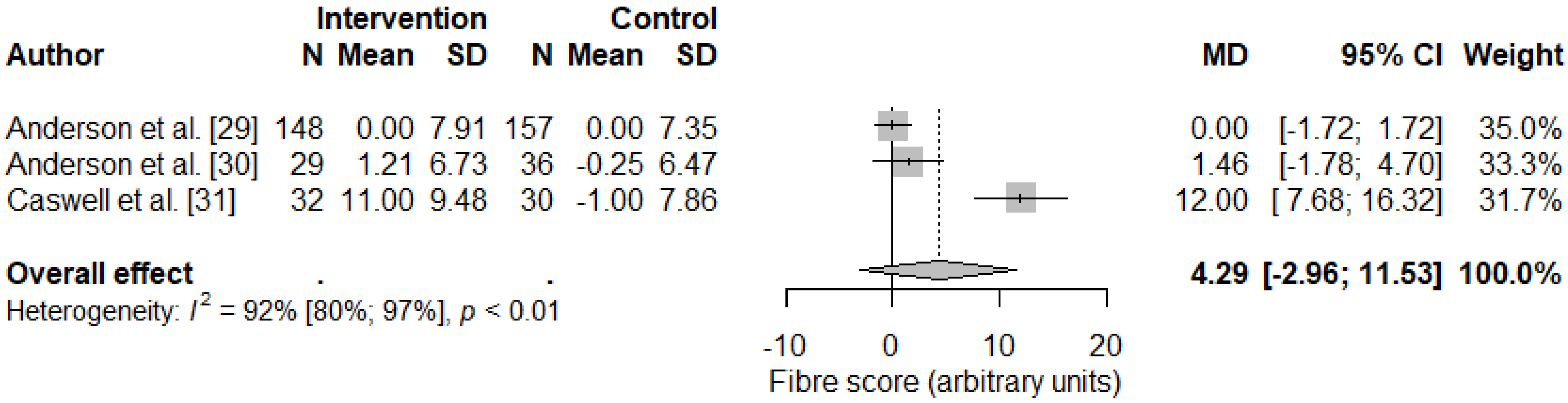

Electronic Supplementary Material 5. Forest plot of the results from random-effects meta-analyses on fibre intake. Data are presented as mean difference (MD) between intervention and control groups with corresponding 95% confidence interval (CI).
