# Supplementary material for: Effectiveness of diet and physical activity interventions amongst adults attending colorectal and breast cancer screening: a systematic review and meta-analysis
Source: Cancer Causes Control. 2020 Nov 8;32(1):13–26. doi: 10.1007/s10552-020-01362-5 (PMC7796884; doi:10.1007/s10552-020-01362-5)
Supplement: Supplementary file 6 — Electronic supplementary material 6 (PDF 32 kb) [file 10552_2020_1362_MOESM6_ESM.pdf]

Electronic supplementary material 6. Results from the sensitivity analyses assuming a within-groups correlation coefficient (*r*) of 0.5 and 0.9 (instead of 0.7) to estimate the change score standard deviation

	Pooled treatment effect (95% CI)	*p*-value	I^2
**Correlation coefficient of 0.5**			
Fibre intake (MD, arbitrary units)	4.1 (-2.9, 11.2)	0.25	87%
Physical activity (SMD)	0.27 (0.08, 0.47)	0.007	5%
Fruit/vegetable intake (SMD)	0.28 (0.04, 0.52)	0.025	29%
**Correlation coefficient of 0.9**			
Fibre intake (MD, arbitrary units)	4.4 (-3.0, 11.6)	0.24	97%
Physical activity (SMD)	0.46 (0.27, 0.64)	<0.001	0%
Fruit/vegetable intake (SMD)	0.49 (-0.04, 1.0)	0.072	89%

95% CI = 95% confidence interval; MD = mean difference; SMD = standardised mean difference
